# Composition, Source Apportionment, and Health Risk of PM_2.5_-Bound Metals during Winter Haze in Yuci College Town, Shanxi, China

**DOI:** 10.3390/toxics10080467

**Published:** 2022-08-11

**Authors:** Lihong Li, Hongxue Qi, Xiaodong Li

**Affiliations:** Department of Chemistry and Chemical Engineering, Jinzhong University, Jinzhong 030619, China

**Keywords:** heavy metals, atmosphere, PMF

## Abstract

The composition, source, and health risks of PM_2.5_-bound metals were investigated during winter haze in Yuci College Town, Shanxi, China. The 24-h PM_2.5_ levels of 34 samples ranged from 17 to 174 μg·m^−3^, with a mean of 81 ± 35 μg·m^−3^. PM_2.5_-bound metals ranked in the following order: Zn > Cu > Pb > As > Ni > Cr (VI) > Cd > Co. The concentrations of 18% As and 100% Cr (VI) exceeded the corresponding standards of the Ambient Air Quality Standards set by China and the WHO. Subsequently, positive matrix factorization analyses revealed that the three major sources of metals were combustion (37.91%), traffic emissions (32.19%), and industry sources (29.9%). Finally, the non-carcinogenic risks for eight metals indicated that only 2.9% of the samples exceeded a threshold value of one, and As accounted for 45.31%. The total carcinogenic risk values for six metals (As, Cd, Co, Cr (VI), Ni, and Pb) were in the range from 10^−6^ to 10^−4^, with Cr (VI) and As accounting for 80.92% and 15.52%, respectively. In conclusion, winter haze in Yuci College Town was characterized by higher metal levels and health risks; among the metals, As and Cr (VI) were probably the main contributors.

## 1. Introduction

Atmospheric haze has attracted considerable attention, especially during winter. Atmospheric fine particulate matter (PM_2.5_) plays an important role in hazy episodes and is the fifth leading cause of death globally after high blood pressure, smoking, diabetes, and hypercholesterolemia [1]. It is significantly associated with the incidence and mortality of bronchitis, asthma, and lung cancer [2]. PM_2.5_ can be attached to a wide variety of chemical contaminants, such as polycyclic aromatic hydrocarbons (PAHs), heavy metals, nitrogen oxides, and emerging pollutants [3]. Some toxicants can be absorbed by the human respiratory system and can affect human health.

Studies on PM_2.5_-bound metals have been performed throughout the world; in some areas, the concentrations of PM_2.5_-bound metals have exceeded the threshold range of the WHO global air quality guidelines [4], such as in Isfahan of Iran [5], Saudi Arabia [6], Kitakyushu of Japan [7], as well as Beijing–Tianjin–Hebei [8], Xi’an [9], Guangzhou [10], and Taiyuan in China [11]. The source apportionment of heavy metals has helped to establish targeted pollution control strategies; for instance, coal burning, industrial pollution, and traffic often have been identified as the major contributors of metals [12,13,14]. In addition, PM_2.5_-bound metals pose health risks to humans [15]. Some metals have been identified as toxic and hazardous air pollutants in China [16] and some metals (e.g., As, Cd, Co, Cr, Ni, and Pb) have been defined by the WHO as carcinogenic to humans [17]. Therefore, a better understanding of the composition, source apportionment, and carcinogenic risk of metals in PM_2.5_ is crucial to protect human health, especially during haze periods.

Yuci College Town is located in the Yuci District, Jinzhong City, Shanxi Province, China, adjacent to the provincial capital of Taiyuan City. It covers an area of approximate 12 km^2^. It is a campus of provincial colleges and universities built by Shanxi Province. Currently, there are nearly ten colleges and universities, such as Taiyuan University of Technology, Shanxi Medical University, Taiyuan Normal University, and Jinzhong University. The total number of teachers and students in Yuci College Town is about 150,000. The campus has experienced frequent heavy air pollution in the past, particularly in winter. For instance, from January 2016 to December 2018, heavy air pollution events occurred 23 times for a total of 88 days in Jinzhong, Shanxi, China [18]. The predominant source of pollution was PM_2.5_, which accounted for 78.3% of the total pollution.

Shanxi Province is well-known for its coal resources in China, and coal mining is considered to be one of the most significant sources of heavy metal contamination. As previously reported, heavy metal pollution from As and its carcinogenic risk to humans have been reported in Shanxi’ mines [19]. Atmospheric PM_2.5_ pollution levels are very important to the health of teachers and students. However, investigations of PM_2.5_-bound heavy metal pollution are lacking in Yuci College Town, Shanxi, China (YCT of China), especially during winter haze periods.

The objectives of the present study were (1) to measure the concentrations of heavy metals (including As, Cd, Co, Cr, Cu, Ni, Pb, and Zn) in PM_2.5_ in YCT of China; (2) to analyze the source apportionment; and (3) to assess the health risks (non-carcinogenic and carcinogenic) of exposure to eight heavy metals, via inhalation exposure, during winter haze periods.

## 2. Experiments and Methods

### 2.1. PM_2.5_ Sample Collection

**Figure 1:**
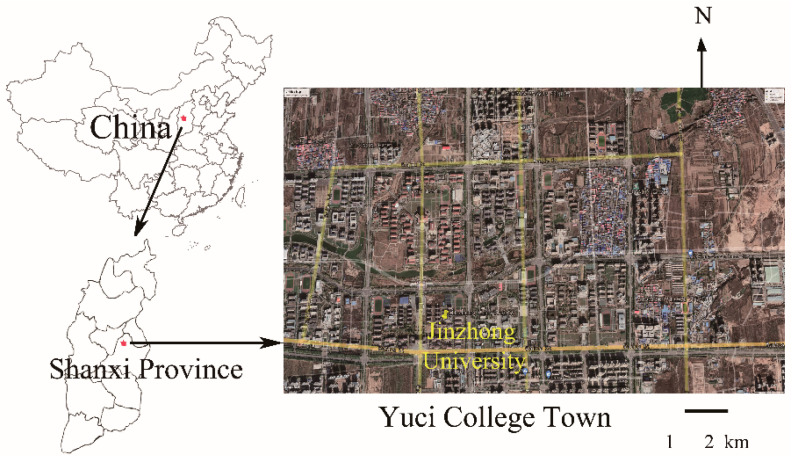
Map of the sampling area in Yuci College Town, Shanxi, China.

Figure 1 describes the location of the sampling site in Jinzhong University (Yuci district, Jinzhong City, Shanxi Province, China). This site is located at the southwest of Yuci College Town as well as multiple campuses and residential areas, representing an urban area. From 3 November 2020 to 9 December 2020, a total of 34 daily PM_2.5_ samples were collected using a medium flow particle sampler and quartz fiber filters at a gas flow rate of 100 L·min^−1^. The sampler was placed 15 m from the ground and surrounded by pollution-free emission sources. The daily 24-h mean of PM_2.5_ was calculated using the gravimetric method [20]. The filters were all baked at 450 °C for 4 h before sampling to remove organic contaminants. Then, the filters were placed in boxes with constant humidity (50% ± 5%) and temperature (24 ± 1 °C) for use. The filters were weighed after sampling, and the sensitivity of the balance was 0.01 mg. Finally, the filters were stored at −20 °C, and pollutants were extracted within 2 months.

### 2.2. Metals Analysis

The PM_2.5_ filters were digested on mixed acid, dissolved using a microwave digestion system, and the concentrations of eight metals (As, Cd, Co, Cr, Cu, Ni, Pb, and Zn) were analyzed by internal calibration using an inductively coupled plasma–mass spectrometer (ICP-MS, Agilent 7700), according to the method of HJ 657-2013 in China [21]. The concentration of Cr (VI) was calculated to be 1/7 of the measured Cr concentration according to the US EPA regional screening levels [22]. Detailed information on the digestion, internal standards (Table S1), and detection limit for metal analysis are shown in the Supplementary Information (SI).

### 2.3. PMF Model

The positive matrix factorization (PMF) model is widely used for source apportionment of PM_2.5_. It can simply transform the input data (multiple pollutant data arranged in matrix form) into a factor profile matrix and a factor contribution matrix. Detailed information was obtained according to the US EPA PMF 5.0 Fundamentals and User Guide [23]. Uncertainties (*Unc*) were calculated using the following equation:

If the concentration was higher than the method detection limit (MDL):

(1) $$U_{nc}=\sqrt{{\left(\mathrm{Error}\;\mathrm{fraction}\times \mathrm{concentration}\right)}^{2}+{\left(0.5\times \mathrm{MDL}\right)}^{2}}$$

If the concentration was less than or equal to the MDL:

(2) $$U_{nc}=\frac{5}{6}\times \mathrm{MDL}$$

In addition, the rate of *Q*_robust_/*Q*_true_ was calculated to determine the optimal number of factors, and bootstrap (BS) and displacement (DISP) analyses were performed to estimate the uncertainties of the PMF model.

### 2.4. Human Health Risk Assessment

Health risks of heavy metals in PM_2.5_ were estimated via the inhalation pathway, as proposed by the US EPA [11]. The exposure concentration (EC) was calculated to assess the carcinogenic risk using Equation (3):

EC = (CA × ET × EF × ED)/AT(3)

where EC is the exposure concentration (μg·m^−3^), CA is the contaminant concentration in air (μg·m^−3^), ET is the exposure time (24 h·d^−1^), EF is the exposure frequency (180 d·y^−1^), ED is the exposure duration (24 y for adults), and AT is the averaging lifetime: for non-carcinogens (24 × 365 × 24 h) and for carcinogens (70 × 365 × 24 h).

The hazard index (HI) is traditionally used to assess the overall non-carcinogenic risk posed by multiple chemicals, and it was hypothesized that all metal risks were additive effects despite existing synergistic effects, the equation for HI is defined as follow:

HI = ∑HQ*_i_* = ∑EC*_i_*/RfC*_i_* (4)

where HQ is the hazard quotient (unitless) and RfC*_i_* is the reference concentration of *i*th heavy metal (μg·m^−3^) for inhalation (Table 1). According to the US EPA [24], if the HI value is less than one, the exposed population is unlikely to experience obvious adverse health effects. In contrast, if the HI value exceeds one, an adverse effect may occur for a specific population.

Carcinogenic risk (CR) is defined as the probability of an individual developing any type of cancer throughout their lifetime owing to exposure to carcinogenic hazards. CR was summarized by inhalation for an individual over a lifetime according to the following equation:

CR = ∑CR*_i_* = ∑IUR × EC*_i_* (5)

where IUR is the inhalation unit risk (μg·m^−3^)^−1^ (Table 1). According to the US EPA [24], a CR lower than 10^−6^ indicates an acceptable level, a CR range of 10^−6^ to 10^−4^ is generally considered a potential risk level, and a CR above 10^−4^ is likely to be harmful to the human body.

### 2.5. Air Mass Backward Trajectory

Backward trajectory was performed using the hybrid single-particle Lagrangian integrated trajectory (HYSPLIT) online model from the National Oceanic and Atmospheric Administration [27]. The start altitude was chosen at three different heights (50, 500, and 1000 m), representing the low, middle, and upper atmosphere, respectively. The global data assimilation system (GDAS) data of 72-h backward trajectories on 25 November 2020 are presented in Figure S1.

### 2.6. Network Data Collection

The consumption of end-use energy, the number of days that reached the air quality standards, and the annual mean concentrations of PM_2.5_ in Shanxi, China, were collected from the Shanxi Statistics Yearbook [28].

## 3. Results and Discussion

### 3.1. Mass Levels of PM_2.5_

The levels of PM_2.5_ are presented in Table S2, and their descriptive statistics are presented in Table 2. The daily PM_2.5_ concentrations of 34 samples ranged from 17 to 174 μg·m^−3^, with a median of 74 μg·m^−3^ and an average concentration of 81 ± 35 μg·m^−3^; 94% of the samples exceeded Grade I (35 μg·m^−3^) of the Chinese ambient air quality standards [29]; none of the samples reached the WHO’s AQG level (15 μg·m^−3^) in 2021 [30]; however, 53% of the samples reached the Grade II standard (75 μg·m^−3^) [29] and Interim Target-1 (75 μg·m^−3^) of the WHO global air quality guidelines [30]. During the sampling period, there were two heavy haze episodes, including 11 to 16 November and 24 to 26 November. During haze episodes, the PM_2.5_ concentrations averaged over 100 μg·m^−3^ and peaked at 127 and 174 μg·m^−3^, respectively. These results indicate that air pollution during winter was severe in this area.

### 3.2. Concentrations of Heavy Metals in PM_2.5_

The levels of eight PM_2.5_-bound heavy metals are presented in Table S2 and their descriptive statistics are presented in Table 2. The total concentration of the eight metals was 235.87 ± 161.88 ng·m^−3^. The daily mean levels of the heavy metals were ranked in the order Zn > Cu > Pb > As > Ni > Cr (VI) > Cd > Co. Zn was the most abundant metal with a mean of 191.87 ± 145.92 ng·m^−3^, followed by Cu (20.04 ± 17.35), Pb (14.95 ± 9.09), As (4.71 ± 2.70), Ni (1.82 ± 1.48), Cr (VI) (1.31 ± 0.77), Cd (0.89 ± 0.86), and Co (0.29 ± 0.38) ng·m^−3^. Moreover, the concentrations of Cd, Ni, and Pb in all samples were lower than the annual values recommended by the WHO [4] and China [29] (Table 2). However, the concentrations of 18% As and 100% Cr (VI) exceeded the Grade Ⅱ standard of the Chinese Ambient Air Quality Standards [29]. There were no corresponding standards for Co, Cu, and Zn. These results are similar to those found in other areas; for instance, the levels of As and Cr (VI) greatly exceeded their corresponding threshold values in 60 cities in China [31].

In recent years, the mean concentrations of the metals in PM_2.5_ have been obtained from China and abroad, and the results of other studies are summarized in Table 3.

For As, the average concentration during the 2020 winter haze periods in YCT of China was lower than that in Changzhi, Shanxi, China [32]. For other provinces in China, the average concentration of As, during the 2020 winter haze episodes in YCT of China, were higher than those in Beijing [33], Chengdu [34], Guangzhou [10], and Lanzhou [35]. Worldwide, it was higher than those in Tˇrinec-Kosmos of Czech Republic [36] and Kitakyushu of Japan [7]. In contrast, the average concentration of As in the 2020 winter haze episodes in YCT of China was lower than those in Yuci and Taiyuan [11] in 2017 within Shanxi Province. For other provinces in China, it was lower than those in Chongqing [37], Handan [38], Xi’an [9], and Xuanwu [10]. For worldwide, it was lower than those in Iasi of Romania [39], Isfahan of Iran [5], Karaj of Iran [40], and Saudi Arabia [6].

For Cr (VI), since total chromium concentrations were only available from the literature in some regions, the concentrations of Cr (VI) were also calculated to be 1/7 of the Cr concentration according to the US EPA regional screening levels [22]. For comparison, the mean concentration of Cr (VI) during the 2020 winter haze periods in YCT of China was higher than those in Tˇrinec-Kosmos of Czech Republic [36], Iasi of Romania [39], Koldata of India [41], Kitakyushu of Japan [7], Saudi Arabia [6], Los Angeles of USA [12], Beijing [33], and Chongqing [37] of China. Conversely, the concentrations of Cr (VI) in the remaining regions were higher than that of YCT in China. It has been suggested that Cr (VI) in PM_2.5_ is very common and is a common air pollution problem worldwide. More stringent measures are required to control Cr pollution in the future.

Overall, the PM_2.5_-bound heavy metal contents were generally high in the YCT of China, with severe As and Cr (VI) contamination in PM_2.5_ during winter haze periods.

### 3.3. Source Apportionment of PM_2.5_-Bound Elements

The source apportionment of eight heavy metals in PM_2.5_ was conducted using the PMF model [23]. Two to six factors were used for each dataset to determine the optimal solutions. Finally, the optimum result with a three-factor solution (*Q*_robust_ = 1141.5, *Q*_true_ = 1255.4, *Q*_robust_/*Q*_true_ = 0.91, 92–100% of BS runs, and no swaps of DISP runs) was selected based on the interpretability of the source profiles and the results of the modeling diagnostics. The correlation coefficient (*R*^2^) values between the predicted data and the input data ranged from 0.69 to 0.92, meaning a good fit of the model.

**Figure 2:**
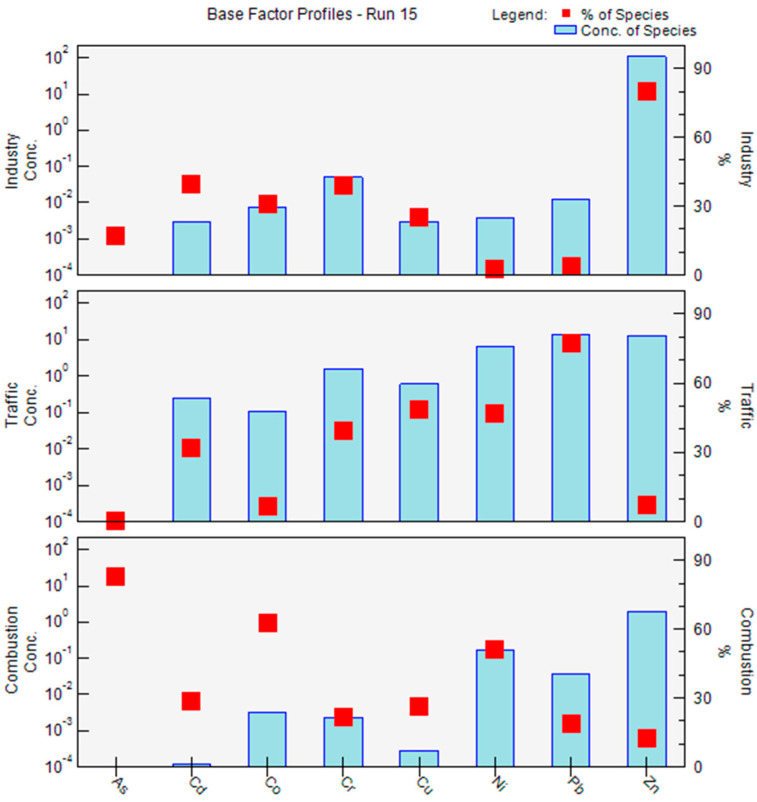
Source profiles obtained by the PMF analyses for Yuci College Town, Shanxi, China. Bars represent mass concentrations and red squares represent contribution percentages from each source factor.

As shown in the factor profile in Figure 2, the PMF resolved three factor profiles, namely, industry, traffic, and combustion, as the three anthropogenic sources contributing to the total PM2.5-bound metals. Factor 1 was associated with high loadings of Cd (39.9%), Cr (VI) (39.2%), and Zn (80.3%). A previous study reported that Cr (VI) originated from the glassmaking industry, while Ni originated from steelworks [45]. Therefore, Factor 1 was identified as an industrial source. Factor 2 accounted for 48.8% and 77.6% of the Cu and Pb concentrations, respectively. Cu and Zn have been documented as surrogates for brake wear [46]. Pb emissions may result from the use of leaded gasoline. Thus, Factor 2 was labeled as a traffic source. Factor 3 demonstrated high loadings of the metals As (82.7%), Co (62.6%), and Ni (50.8%). These elements are all related to coal combustion [47], and Ni is also typical of oil combustion [14]. Therefore, Factor 3 was regarded as “combustion sources”, which included emissions from oil and coal combustion.

**Figure 3:**
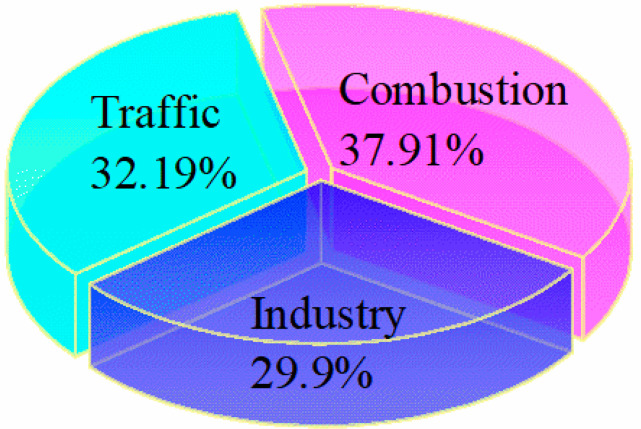
Contributions of identified sources to PM_2.5_-bound metal based on the PMF model in Yuci College Town, Shanxi, China.

Overall, three major sources of PM_2.5_-bound heavy metals were characterized based on the PMF analyses. As shown in Figure 3, combustion was still the largest contributor to PM_2.5_-bound heavy metals (37.91%) in Jinzhong, China. The contribution of traffic emissions (32.19%) was ranked second, just higher than the industry source (29.9%).

Coal combustion was a main contributor that cannot be ignored. Shanxi is a famous coal province in China [48], and coal mining is considered one of the most important sources of heavy metal pollution [49]. More attention should be paid to the burning of coal for heating in winter, and the use of clean energy should be further increased.

The second contribution of traffic sources resulted from the increase in car ownership year by year. For example, the number of cars in Shanxi Province increased from 3.76 million in 2013 to 7.61 million in 2020 [28]. However, in the following years, this situation is expected to improve because of the continuous investment of new energy vehicles [50].

**Figure 4:**
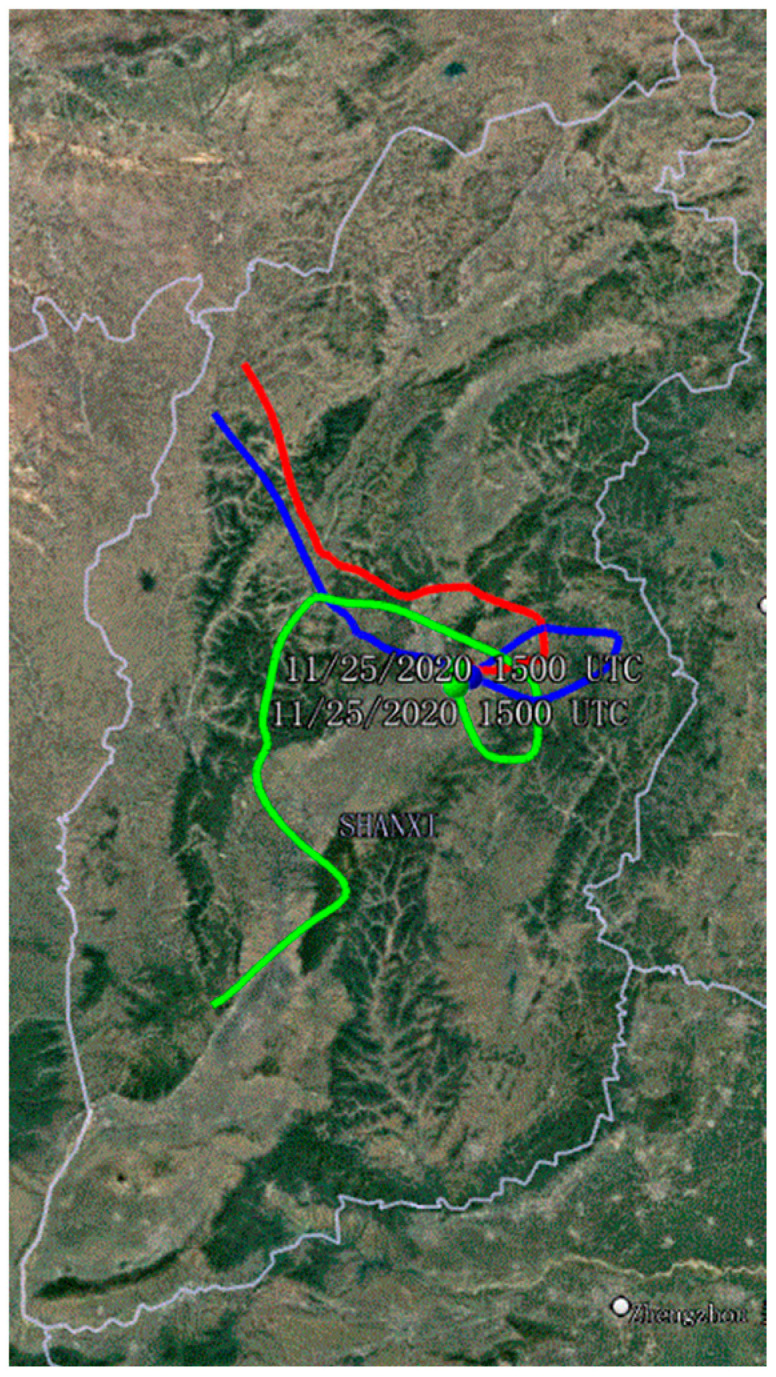
The 72-h back trajectories on 25 November 2020 in Yuci College Town, Shanxi, China. The red color line represents the height of 50 m; blue line: 500 m; green line: 1000 m.

In fact, the formation of PM_2.5_ is very complex, and they involve adverse meteorological conditions, local emission accumulation, and regional transport, etc. [51]. Industrial sources of heavy metals in PM_2.5_ were likely to be regional transport because there were almost no industrial pollution sources around the sampling sites. Evidence of regional transport resulted from the analysis of the reverse trajectory. As shown, 72-h air mass backward trajectories occurred in haze episodes on 25 November 2020 (Figure 4); the results of 50 and 100 m were basically the same, and the air masses passed through Xinzhou-Taiyuan and reached Yuci after a roundabout in Yangquan. At 1000 m, the air mass passed through Linfen–Lvliang–Taiyuan, made a detour through Yangquan before reaching Yuci. These results indicated that most of the long-distance air masses were influenced by northwest winds and reached YTC of China through several industrial cities. This also indirectly suggested that the industrial pollution in YTC might come from regional transport rather than local sources. Due to the implementation of air pollution control policies and the shift of industrial production, industrial pollution is no longer the main source of pollution in YTC of China.

### 3.4. Human Health Risk Assessment

#### 3.4.1. Non-Carcinogenic Risk Assessment

**Figure 5:**
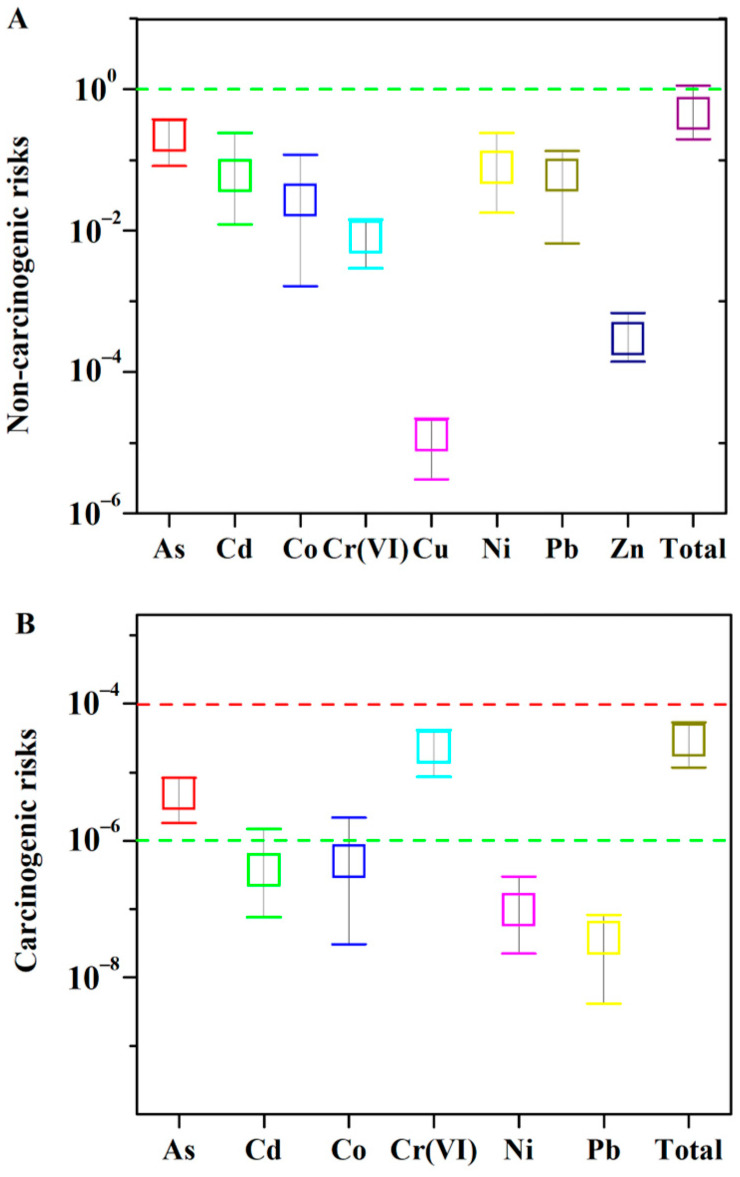
Non-carcinogenic (**A**) and carcinogenic (**B**) risks of exposure to PM_2.5_-bound metals estimated via the inhalation route in Yuci College Town, Shanxi, China (*n* = 34).

The non-carcinogenic risks of the eight PM_2.5_-bound metals were calculated via the inhalation route in YCT of China (Figure 5). HI values ranged from 0.052 to 1.12 in 34 samples with a median of 0.30 and a mean of 0.34 ± 0.21. In this study, 2.9% of the samples exceeded an acceptable threshold of one. Among the eight metals, As accounted for 45.31% of the entire HI value, which demonstrated that the metal As was probably the main contributor to non-carcinogenic risk, while the contributions of the remaining metals were negligible. It can be concluded that heavy metals in PM_2.5_ have low non-carcinogenic risks to the public in YCT of China.

#### 3.4.2. Cancer Risk Assessment

The carcinogenic risk of exposure to six metals (As, Cd, Co, Cr, Ni and Pb) was estimated via the inhalation route and are presented in Figure 4. They ranged from 7.56 × 10^−6^ to 5.36 × 10^−5^, with an average value of (2.29 ± 1.30) × 10^−5^. The carcinogenic risk values all ranged from 10^−6^ to 10^−4^. Among the six metals, Cr (VI) was the main contributor to carcinogenic risk, accounting for 80.92 ± 6.20% of the total CR, and As accounted for 15.52 ± 5.42% of the total CR. These results demonstrated that the metals As and Cr (VI) were probably the main contributors to carcinogenic risk, while the contribution of the remaining metals was negligible. These values are lower than the total CR of metals in PM_2.5_ reported in Taiwan, China [52], but are higher than the total CR reported in Changzhi, China (10.31 × 10^−6^) [32]. These results are consistent with previous studies that indicated the CR also mainly resulted from the contribution of Cr in Shenzhen [53], Taiyuan and Yuci [11], and Changzhi [32] in China. In addition, Cr (VI) also contributed to the highest potential years of life lost in most cities, with a proportion of 72.7% across 60 cities in China [31].

In summary, the non-carcinogenic risks of heavy metals were negligible in PM_2.5_ in YCT of China. However, more attention should be paid to the carcinogenic risks of heavy metals, especially As and Cr (VI).

### 3.5. Policy Implication

A series of strict control measures have been implemented to prevent and control air pollution in China, and the PM_2.5_ levels have declined since 2013. For instance, China’s “Action Plan for the Prevention and Control of Air Pollution” was proclaimed in 2013; the “Blue Sky Protection” campaign was enacted in 2018; the 14th “Five-Year Plan for Modern Energy System” was issued in 2022; and the “Opinions on Further Strengthening the Prevention and Control of Heavy Metal Pollution” was issued in March 2022, which set two goals for 2025 and 2035. By 2025, the emissions of key heavy metal pollutants from key industries will be reduced by 5 percent as compared with those in 2020. By 2035, a heavy metal pollution prevention and control system and long-term mechanism will be established to comprehensively improve the ability to monitor environmental pollution, to control heavy metal pollution, and to prevent environmental risks. In addition, Shanxi has implemented many policies, such as promoting coal energy transformation, developing emerging industries, limiting the traffic volume in winter by odd or even days, and operating new energy vehicles.

**Figure 6:**
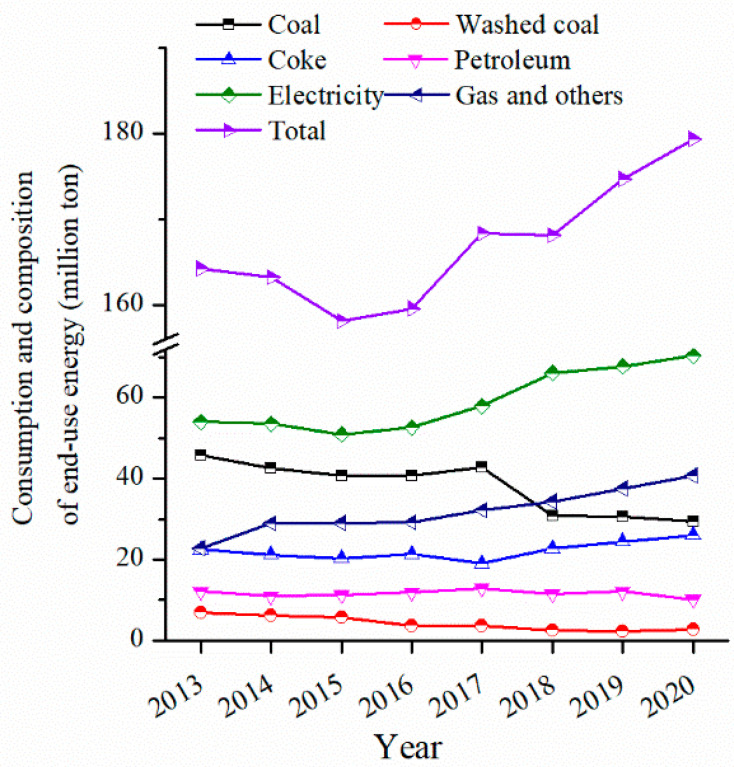
The composition of energy consumption in recent years in Shanxi Province, China.

The implementation of coal banning was very effective in controlling air pollution during winter. For instance, the proportion of coal consumption in Shanxi Province decreased annually, while the proportion of electricity and natural gas increased. As shown in Figure 6 (original data were presented in Table S3), from 2013 to 2020, the proportion of coal consumption decreased from 27.92% to 16.39%, while electricity increased from 32.90% to 39.33% and natural gas increased from 13.89% to 22.72%.

**Figure 7:**
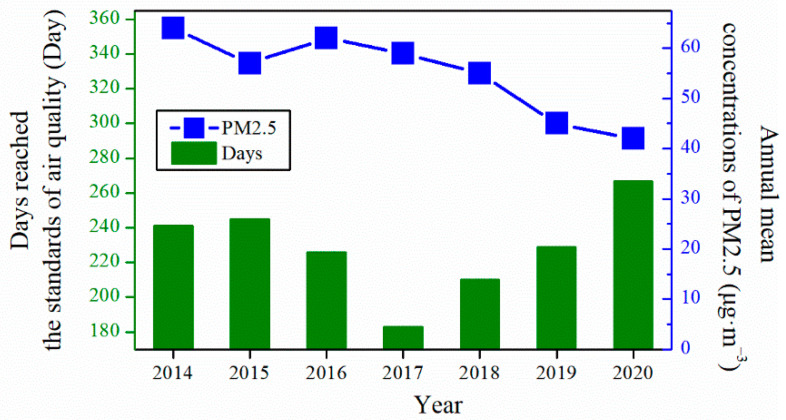
The number of days that reached the air quality standards in recent years and the annual mean concentrations of PM_2.5_ in Jinzhong, Shanxi Province, China.

Air quality has improved significantly, owing to a series of strict control policies in China. The number of days that reached the air quality standards gradually increased, while the average annual PM_2.5_ level gradually decreased. As shown in Figure 7 (the original data are listed in Table S4), from 2014 to 2020, in Jinzhong, Shanxi Province, China, the number of days that reached the air quality standards increased from 241 to 267 days, while the annual mean concentrations of PM_2.5_ decreased from 64 to 42 μg·m^−3^. These results indicate that Shanxi’s energy transformation has achieved initial effects and will continue to control coal consumption and further increase the use of clean energy to win the battle for a blue sky in the future.

### 3.6. Limitations

First, there are two main types of health risk assessments of PM_2.5_-bound heavy metals. One type of health risk assessment is based solely on one exposure route, i.e., the inhalation route, while the other type of health risk assessments considers three exposure routes: inhalation, dermal, and oral intake. The former type was applied for health risk assessments in the industrial cities of Iran [40], Kolkata of India [41], Beijing of China [13], and Tianjin of China [38]. The latter type was used in Saudi Arabia [6], Hebei of China [54], and Shenzhen of China [53]. However, which is more representative? The models and parameters of health risk assessment should be optimized for specific populations and regions. Second, the traditional approach to heavy metals inherently hypothesizes that the carcinogenic risks of all metals are additive effects in a mixture. However, the synergistic or antagonistic effects may also occur during metabolism. For instance, three joint effects (synergistic, antagonistic, or additive effects) of the toxicity were all observed in the different component metal mixtures (Cd, Cr, Cu, Hg, Mn, Ni, Pb, and Zn) [55]. The mixtures of Cd + Pb [56], Cd + Ni [57], and Zn + Al [46] have synergistic effects [58], while Cd + Cu [59] and Cd + Zn [60] mixtures have antagonistic effects. In addition, heavy metals are only a small part of the PM_2.5_, which is also composed of other substances, such as organic matter, nitrate, sulfate, ammonium, elemental carbon, and chloride [61]. In future research, the above insufficiency should be overcome to assess the risk to human health more accurately.

## 4. Conclusions

In this study, the levels of eight metals in PM_2.5_ were detected during the 2020 winter haze periods in YCT of China. The 24-h PM_2.5_ levels of 34 samples ranged from 17 to 174 μg·m^−3^, with a mean of 81 ± 35 μg·m^−3^. The PM_2.5_-bound heavy metals ranked in the following order: Zn > Cu > Pb > As > Ni > Cr (VI) > Cd > Co. A total of 18% and 100% of the concentrations assessed in the samples for As and Cr, respectively, exceeded the corresponding threshold values of the Chinese Ambient Air Quality Standards and the WHO Global Air Quality Guidelines; the levels of As and Cr (VI) were also higher than those in some areas worldwide. Overall, higher PM_2.5_ levels were found in this area, suffering from severe As and Cr (VI) contamination during winter haze periods.

Based on the PMF model, combustion was the largest contributor to PM_2.5_-bound heavy metals (37.91%), followed by traffic emissions (32.19%) and industrial sources (29.9%). Finally, the potential health risks were estimated through exposure by the inhalation route. The non-carcinogenic risks of PM_2.5_-bound heavy metals were negligible, but the carcinogenic risk values were all within the potential level (10^−6^–10^−4^), and both As and Cr (VI) were the main contributors. Therefore, more attention should be paid to the prevention and control of As and Cr (VI) pollution in the environment. By reducing coal burning and using clean energy in winter, we will continue to reduce industrial source pollution, thereby making a positive contribution to reduce the occurrence of winter haze periods.

## Figures and Tables

**Table 1:** Toxicological parameters of the heavy metals used for health risk assessment via the inhalation route.

	Contaminants	Class ^a^	RfC*_i_* ^b^	IUR ^b^
		WHO	μg·m^−3^	(μg·m^−3^)^−1^
As	Arsenic	1	0.015	0.0043
Cd	Cadmium	1	0.01	0.0018
Co	Cobalt	2B	0.006	0.009
Cr (VI)	Chromium	1	0.1	0.084
Cu	Copper	- ^c^	1000 ^d^	-
Ni	Nickel	2B	0.014	0.00026
Pb	Lead	2B	0.15 ^e^	0.000012
Zn	Zinc	-	300	-

^a^ Class: Agents classified by IARC the monographs adapted with permission from Ref. [17]. 2021, International Agency for Research on Cancer. ^b^ RfC*_i_*: reference concentration of *i*th heavy metal; IUR: inhalation unit risk, with their values from the US EPA: Regional screening levels (RSLs)—Generic Tables [22]. ^c^ Not reported. ^d^ Data from the literature [25]. ^e^ Data from the literature [26].

**Table 2:** Statistical description of the daily PM_2.5_ mass (μg·m^−3^) and the concentrations of its metals (ng·m^−3^) during winter in 2020 at Yuci College Town, Shanxi, China (*n* = 34).

	PM_2.5_	As	Cd	Co	Cr (VI)	Cu	Ni	Pb	Zn	Sum
Min	17	0.43	0.04	0.01	0.39	0.69	0.22	1.04	0.71	27.92
Median	74	4.22	0.68	0.15	0.99	12.82	1.48	15.19	164.02	196.46
Max	174	11.36	4.88	1.61	2.91	67.15	6.82	40.52	823.39	905.26
Mean	81	4.71	0.89	0.29	1.31	20.04	1.82	14.95	191.87	235.87
SD ^a^	35	2.70	0.86	0.38	0.77	17.35	1.48	9.09	145.92	161.88
WHO guideline value ^b^	25	6.6	5	-	0.25	-	25	500	-	
Grade II threshold ^c^	75	6	5	- ^d^	0.025	-	-	500	-	

^a^ SD: standard deviation. ^b^ WHO global air quality guidelines adapted with permission from Ref. [4]. 2005, World Health Organization. ^c^ Annual average concentrations of the Chinese ambient air quality standards [29]. ^d^ Not reported.

**Table 3:** A comparison of the results from other studies regarding the mean concentrations (ng·m^−3^) of PM_2.5_-bound heavy metals in urban regions.

Country	Areas	Year	PM_2.5_	As	Cd	Co	Cr (VI)	Cu	Ni	Pb	Zn	References
Czech Republic	Třinec-Kosmos	2020	28	1.3	0.26	0.05	1.1/7 ^d^	4.6	0.81	11	34	[36]
Romania	Iasi	2016	20	**5.70**	0.33	0.09	1.78/7	7.70	24.3	1.99	33.9	[39]
India	Kolkata	2019	111.7	-	1.2	0.3	6.9/7	15	8.1	36	370	[41]
Iran	Isfahan	2015	- ^a^	**32.47** ^d^	5.77	-	**57.4/7**	13.76	7.43	46.72	-	[5]
Iran	Karaj	2019	67	**32.1**	84	-	**49.5/7**	203	60.8	133	242	[40]
Japan	Kitakyushu	2019	21.3	1.4	-	-	3.0/7	3.6	3.3	10.5	29.5	[7]
Saudi Arabia		2020	-	**83**	17	-	8/7	9	10	119	31	[6]
USA	Los Angeles	2018	13.8	-	6	1	3/7	10	3	5	10	[12]
China	Beijing	2019	-	4.02	-	-	1.79/7	7.37	0.77	21.13	78.99	[33]
	Chengdu	2018	113.2	4.5	-	-	-	7.5	7.7	21.9	60.8	[34]
	Chongqing	2019	97.1	**7.56**	-	-	4.29/7	15.83	1.39	37.93	94.22	[37]
	Guangzhou	2017	55	4.39	0.74	0.53	**10.1/7**	16.37	5.72	25.52	127.31	[10]
	Guilin (haze)	2017	144	-	19.0	-	**11.5/7**	17.4	-	78.8	300.7	[42]
	Handan	2017	-	**11.94**	2.74	-	**11.1/7**	23.17	2.11	104.3	286.9	[43]
	Hefei	2017	81	-	-	-	**10/7**	11.29	-	12.64	273.5	[44]
	Lanzhou	2018	73	3	1	1.3	-	29	-	407	-	[35]
	Xi’an	2016	50.1	**117.2**	16.3	-	**343/7**	-	11.3	35.0	267.1	[9]
	Xuanwu	2016	61.1	6.44	1.88	0.29	**77.5/7**	20.99	3.73	54.72	212.76	[10]
Shanxi,	Changzhi	2018	56.1	4.9	0.7	0.2	**14.3/7**	7.8	4.2	30.8	82.3	[32]
China	Taiyuan	2017	-	**8.15**	1.07	1.20	**29.9/7**	29.56	12.69	94.36	230.57	[11]
	Yuci	2017	-	**9.45**	1.12	0.70	**11.7/7**	14.66	3.56	91.29	263.26	[11]
	Yuci	2020	80.65	4.71	0.89	0.29	1.31	20.04	1.82	14.95	191.87	This study
WHO guideline value ^b^			25	6.6	5	-	0.25	-	25	500	-	
Grade II threshold ^c^			75	6	5	-	0.025	-	-	500	-	

^a^ Not reported. ^b^ WHO global air quality guidelines adapted with permission from Ref. [4]. 2005, World Health Organization. ^c^ Annual average concentrations of the Chinese ambient air quality standards [29]. ^d^/7: The concentration of Cr (VI) was calculated to be 1/7 of the Cr concentration according to the US EPA regional screening levels [22]. Values higher than those in Yuci College Town are shown in bold.

## Data Availability

Not applicable.

## Supplementary Materials

The following supporting information can be downloaded at: https://www.mdpi.com/article/10.3390/toxics10080467/s1. Text: Detailed description of chemical analyses; Table S1: Isotopes of heavy metals, corresponding internal standards, and detection limit for ICP-MS analysis; Table S2: PM_2.5_ mass (μg·m^–3^) and its metals concentrations (ng·m^−3^) during winter in 2020 in the college town of Shanxi Province, China (*n* = 34); Table S3: The consumption, composition, and their proportion (%) of end-use energy (million ton) in recent years in Shanxi Province, China; Table S4: The days reached the standards of air quality in recent years and the annual mean concentrations of PM_2.5_ (μg·m^−3^) in Jinzhong, Shanxi Province, China; Figure S1. The 72-h back trajectories on 25 November 2020 in Yuci College Town, Shanxi, China. References [21,62,63,64,65,66,67,68,69] are cited in the supplementary materials.
