# Supplementary material for: Composition, Source Apportionment, and Health Risk of PM2.5-Bound Metals during Winter Haze in Yuci College Town, Shanxi, China
Source: Toxics. 2022 Aug 11;10(8):467. doi: 10.3390/toxics10080467 (PMC9415865; doi:10.3390/toxics10080467)
Supplement: Supplementary file 1 [file toxics-10-00467-s001.zip › toxics-1811149-supplementary.pdf]

Supplementary Files

1. Detailed description of chemical analyses

$PM_{2.5}$-bound metals, included As, Cd, Co, Cr, Cu, Ni, Pb, and Zn. were analyzed using an inductively coupled plasma-mass spectrometer (ICP-MS, Agilent 7700). Multi-element standards were obtained from Agilent, an internal standard solution for ICP-MS analysis, included Li, Sc, Ge, Y, Rh, In, Re and Bi (CFGG-163175-02-01), was obtained from the National Standard Samples Website, and high purity nitric acid and hydrofluoric acid were purchased from Beijing Chemical Reagent Factory (Beijing, China). Analytical methods are presented below.

Digestion

The half filter was cut into small pieces with ceramic scissors and put into a digestion cell. The digestion was carried out on a microwave digestion system (WX-6000, PreeKem Scientific Instruments Co., Ltd.) with a digestion solution of 10 mL mix of nitric acid and Hydrochloric acid. The program for microwave digestion was heated to 100 °C within 5 min, held at 100 °C for 2 min; then to 150 °C within 5min, and held at 150 °C for 3 min. Final to 200 °C within 5 min, and held at 200 °C for 15 min. After cooling, 10 mL of triple-distilled water was injected into the tubes and set for 30 min, and then the solution was filtered to remove impurities and diluted by triple-distilled water to 20 mL, finally stored at 4 °C for analysis.

Instrumental analysis

Internal calibration was used to quantify the target metals, all samples were spiked with internal standard solution, the isotope of each element and its corresponding internal

standard were selected and presented in **Table S1**[1]. The concentrations of metal analysis were conducted on an ICP-MS. The RF power, S/C temperature, and analyzer pressure were set at 1550 W, 2 °C, and 1×10^{-4} Pa, respectively. Argon was used as carrier gas and circuit flow was at 1 L/min.

Table S1. Isotopes of heavy metals, corresponding internal standards, and detection limit for ICP-MS analysis

Elements	Isotope [a]	Internal standard [a]	Detection limit ($ng\cdot m^{-3}$)
As	75	^{74}Ge	0.4
Cd	111	^{115}In	0.03
Co	59	^{74}Ge	0.03
Cr	52	^{45}Sc	0.3
Cu	63	^{74}Ge	0.6
Ni	60	^{74}Ge	0.2
Pb	208	^{209}Bi	0.6
Zn	66	^{74}Ge	3

[a]The data were obtained from the National Environmental Protection Standards of China (HJ 657-2013)[1].

Quality assurance and quality control

The detect limitation for eight metals were presented at Table S1. Moreover, solvent blanks, standard reference materials were processed three duplicates for Quality assurance and quality control. As a result, the concentrations of heavy metals in blanks were not detected, the recoveries of target metals were in the range of 95.3% – 108.2%, and their relative deviation were < 6% for all batch treatments. In addition, each batch of 10 samples was typically checked with at least one calibration standard solution and the relative

differences between the calibration curve and the checks were all within 10%.

The performance of the instruments was checked with a calibration standard every 10 samples, and the relative differences between the calibration curve and the daily checks were all within 20%. Solvent blank, matrix blank (control soil), matrix spike, and matrix spike duplicate were processed for every 10 samples. And the recoveries of target metals were in the range of 97.6% – 106.2%. External calibration was used to quantify the target metals, and the eight calibration standards ranging from 1 to 100 mg/L of each metal were prepared by serial dilution from the stock solution (10 mg/mL) using 2% nitric acid. The recoveries of the metals spiked in the control sediment were 78%–129%.

Table S2. $PM_{2.5}$ mass ($\mu g \cdot m^{-3}$) and its metals concentrations ($ng \cdot m^{-3}$) during winter in 2020 in the college town of Shanxi Province, China (n = 34)

No.	Date	$PM_{2.5}$	As	Cd	Co	Cr (VI)	Cu	Ni	Pb	Zn	Total
1	11.3	55	5.49	1.87	0.58	2.08	22.56	1.87	17.73	353.93	406.11
2	11.4	71	7.42	2.12	0.60	2.81	40.55	4.27	24.10	823.39	905.26
3	11.5	102	11.36	4.88	1.43	2.91	43.44	6.82	40.52	416.51	527.87
4	11.6	73	5.94	1.35	1.61	2.32	25.66	5.40	34.04	218.22	294.54
5	11.7	63	2.35	0.76	0.93	0.54	12.36	1.72	9.10	231.33	259.09
6	11.8	56	3.61	0.47	0.53	1.06	9.37	2.25	17.15	140.77	175.21
7	11.9	60	2.93	0.44	0.51	0.82	13.27	2.25	6.29	182.69	209.2
8	11.1	97	4.36	0.67	0.36	0.92	21.01	1.72	18.82	196.51	244.37
9	11.11	114	5.81	0.69	0.21	0.75	12.03	1.17	16.39	144.13	181.18
10	11.12	114	5.96	0.95	0.61	1.20	22.19	3.26	22.55	147.8	204.52
11	11.13	97	4.23	0.76	0.13	0.74	20.44	1.93	7.53	214.99	250.75
12	11.14	119	2.50	0.52	0.24	0.76	6.16	1.18	7.11	136.7	155.17
13	11.15	89	2.83	0.67	0.02	0.91	8.53	1.79	9.64	274.31	298.7
14	11.16	127	4.18	0.25	0.02	0.60	6.63	0.99	2.02	172.7	187.39
15	11.17	21	1.44	0.07	0.05	0.45	0.69	0.51	1.04	164.32	168.57
16	11.21	37	2.31	0.62	0.14	0.39	5.40	1.32	2.98	141.72	154.88
17	11.22	70	4.21	0.44	0.01	0.75	6.95	0.33	4.17	163.02	179.88
18	11.23	68	5.21	0.93	0.11	1.99	63.70	1.72	18.89	128.59	221.14
19	11.24	128	10.88	0.82	0.17	1.95	36.28	3.09	16.13	182.31	251.63
20	11.25	174	4.53	0.54	0.18	0.96	7.53	1.45	15.46	121.11	151.76
21	11.26	131	5.73	1.03	0.17	2.55	44.72	0.51	17.58	170.88	243.17
22	11.27	59	5.75	0.56	0.04	1.05	7.99	0.68	15.20	88.24	119.51
23	11.28	68	1.24	0.45	0.08	0.79	9.47	0.47	15.17	109.34	137.01
24	11.29	103	5.95	1.10	0.09	2.51	41.66	1.33	30.12	86.07	168.83
25	11.3	46	2.88	0.17	0.03	0.59	13.54	0.43	5.27	20.63	43.54
26	12.1	75	1.63	0.20	0.01	0.74	10.42	1.09	13.12	0.71	27.92
27	12.2	94	3.20	0.50	0.09	1.07	15.21	0.66	19.69	41.81	82.23
28	12.3	17	0.43	0.04	0.03	0.64	5.69	0.22	6.93	22.96	36.94
29	12.4	48	4.19	0.94	0.13	1.65	7.30	4.04	10.33	215.86	244.44
30	12.5	80	7.12	0.75	0.02	1.39	25.12	0.95	13.45	294.95	343.75
31	12.6	95	8.16	1.87	0.15	2.43	67.15	1.50	27.94	372.3	481.5
32	12.7	43	2.20	0.71	0.24	1.01	2.57	1.72	10.18	149.59	168.22
33	12.8	44	3.62	0.67	0.06	0.97	5.28	0.69	13.38	163.72	188.39
34	12.9	104	10.65	1.29	0.15	2.38	40.41	2.38	18.15	231.54	306.95

Table S3. The consumption, composition, and their proportion (%) of end-use energy (million ton) in recent years in Shanxi Province, China

Year	Coal	Washed coal	Coke	Petroleum	Electricity	Gas and others	Total	References
2013	45.8666	6.9094	22.5186	12.1254	54.0386	22.8122	164.2708	[2]
2014	42.4682	6.1540	21.1541	10.9299	53.5868	28.9590	163.2519	[3]
2015	40.7302	5.8018	20.2386	11.3328	50.9618	29.0677	158.1329	[4]
2016	40.8313	3.6983	21.3523	11.8801	52.6514	29.1696	159.5830	[5]
2017	42.8102	3.6236	19.0272	12.8570	57.8891	32.1608	168.3679	[6]
2018	30.8398	2.4629	22.7407	11.4748	66.2480	34.3566	168.1228	[7]
2019	30.5186	2.3585	24.3858	12.0558	67.8286	37.5838	174.7311	[8]
2020	29.3988	2.5966	25.9865	10.0944	70.5441	40.7518	179.3722	[9]
2013	27.92	4.21	13.71	7.38	32.90	13.89	100	
2014	26.01	3.77	12.96	6.70	32.82	17.74	100	
2015	25.76	3.67	12.80	7.17	32.23	18.38	100	
2016	25.59	2.32	13.38	7.44	32.99	18.28	100	
2017	25.43	2.15	11.30	7.64	34.38	19.10	100	
2018	18.34	1.46	13.53	6.83	39.40	20.44	100	
2019	17.47	1.35	13.96	6.90	38.82	21.51	100	
2020	16.39	1.45	14.49	5.63	39.33	22.72	100	

Table S4. The days reached the standards of air quality in recent years and the annual mean concentrations of $PM_{2.5}$ ($\mu g \cdot m^{-3}$) in Jinzhong, Shanxi Province, China

Year	Sources	Days	$PM_{2.5}$	Internet addresses
2014	Table 19-77	241	64	http://tjj.shanxi.gov.cn/tjsj/tjnj/nj2015/indexch.htm
2015	Table 19-72	245	57	http://tjj.shanxi.gov.cn/tjsj/tjnj/nj2016/indexch.htm
2016	Table 19-72	226	62	http://tjj.shanxi.gov.cn/tjsj/tjnj/nj2017/indexch.htm
2017	Table 19-71	183	59	http://tjj.shanxi.gov.cn/tjsj/tjnj/nj2018/indexch.htm
2018	Table 19-65	210	55	http://tjj.shanxi.gov.cn/tjsj/tjnj/nj2019/zk/indexch.htm
2019	Table 19-71	229	45	http://tjj.shanxi.gov.cn/tjsj/tjnj/nj2020/zk/indexch.htm
2020	Table 19-59	267	42	http://tjj.shanxi.gov.cn/tjsj/tjnj/nj2021/zk/indexch.htm

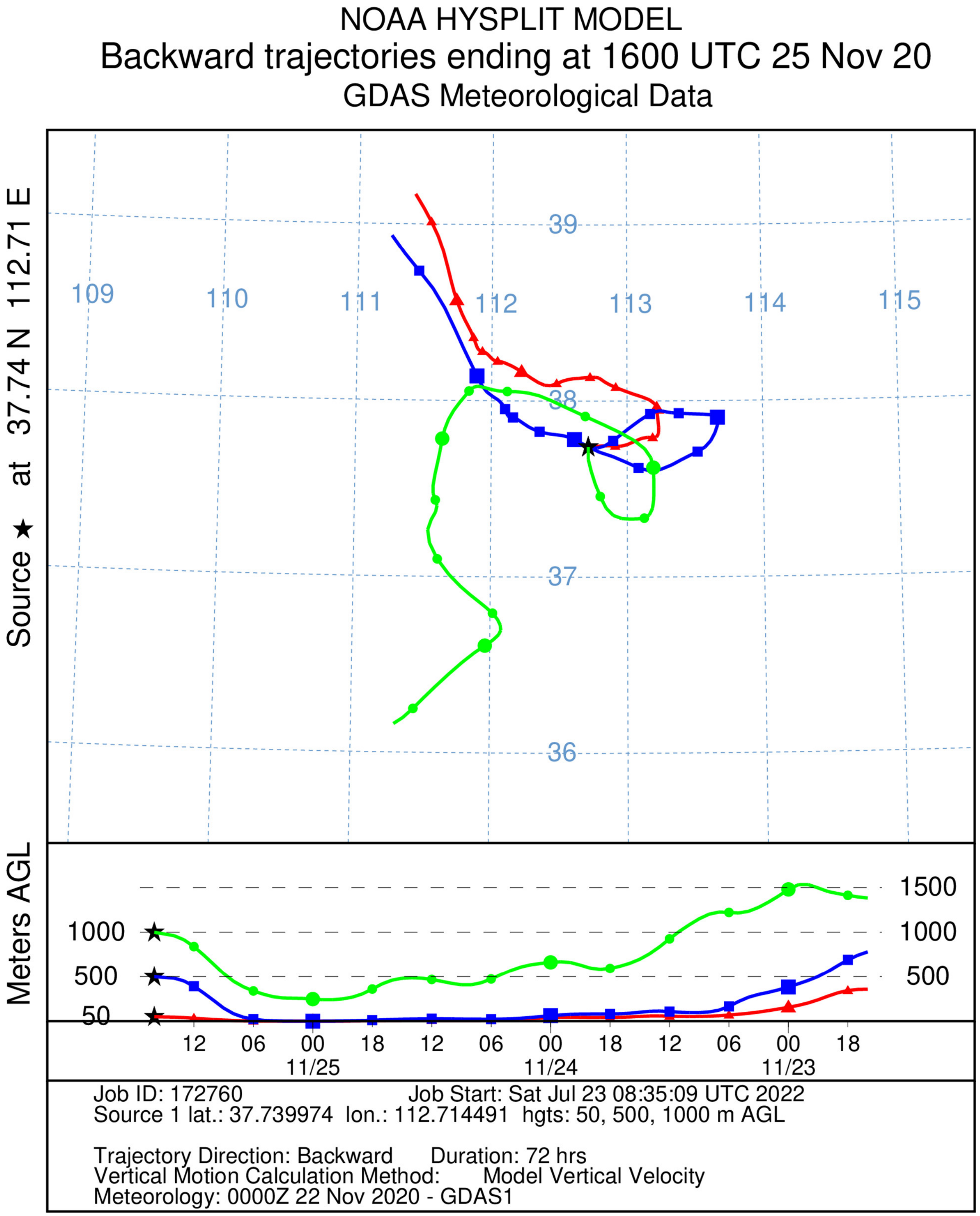

Figure S1. The 72-h back trajectories on 25 November 2020 in Yuci College Town, Shanxi, China.

References

1. NEPSC. *Ambient air and stationary sourc emission: Determination of metals in ambient particulate matter–Inductively coupled plasma/mass spectrometry (ICP-MS) (in Chinese)*; National Environmental Protection Standards of China: Beijing, China, 2013.
2. SSY. *Table 6-15: Consumption and composition of end-use energy in 2013*; Shanxi Statistics Yearbook: http://tjj.shanxi.gov.cn/tjsj/tjnj/nj2014/html/njcx.htm, 2014.
3. SSY. *Table 6-13: Consumption and composition of end-use energy in 2014*; Shanxi Statistics Yearbook: http://tjj.shanxi.gov.cn/tjsj/tjnj/nj2015/indexch.htm, 2015.
4. SSY. *Table 6-13: Consumption and composition of end-use energy in 2015*; Shanxi Statistics Yearbook: http://tjj.shanxi.gov.cn/tjsj/tjnj/nj2016/indexch.htm, 2016.
5. SSY. *Table 6-13: Consumption and composition of end-use energy in 2016*; Shanxi Statistics Yearbook: http://tjj.shanxi.gov.cn/tjsj/tjnj/nj2017/indexch.htm, 2017.
6. SSY. *Table 6-13: Consumption and composition of end-use energy in 2017*; Shanxi Statistics Yearbook: http://tjj.shanxi.gov.cn/tjsj/tjnj/nj2018/indexch.htm, 2018.
7. SSY. *Table 6-13: Consumption and composition of end-use energy in 2018*; Shanxi Statistics Yearbook: http://tjj.shanxi.gov.cn/tjsj/tjnj/nj2019/zk/indexch.htm, 2020.
8. SSY. *Table 6-14: Consumption and composition of end-use energy in 2019*; Shanxi Statistics Yearbook: http://tjj.shanxi.gov.cn/tjsj/tjnj/nj2020/zk/indexch.htm, 2020.
9. SSY. *Table 6-13: Consumption and composition of end-use energy in 2020*; Shanxi Statistics Yearbook: http://tjj.shanxi.gov.cn/tjsj/tjnj/nj2021/zk/indexch.htm, 2021.
